# Frequency of Human CD45+ Target Cells is a Key Determinant of Intravaginal HIV-1 Infection in Humanized Mice

**DOI:** 10.1038/s41598-017-15630-z

**Published:** 2017-11-10

**Authors:** Philip V. Nguyen, Jocelyn M. Wessels, Kristen Mueller, Fatemeh Vahedi, Varun Anipindi, Chris P. Verschoor, Marianne Chew, Alexandre Deshiere, Uladzimir Karniychuk, Tony Mazzulli, Michel J. Tremblay, Ali A. Ashkar, Charu Kaushic

**Affiliations:** 1 McMaster Immunology Research Centre, Michael G. DeGroote Centre for Learning and Discovery, McMaster University, Hamilton, Ontario Canada; 2 Department of Pathology and Molecular Medicine, McMaster University, Hamilton, Ontario Canada; 3 Department of Medical Biology, Laval University, Quebec City, Quebec, Canada; 4 Public Health Laboratories, Public Health Ontario, Toronto, Ontario Canada; 5 Mount Sinai Hospital, Department of Microbiology, Toronto, Ontario Canada; 6 University Health Network, Toronto, Ontario Canada; 7 Present Address: Vaccine and Infectious Disease Organization-International Vaccine Centre (VIDO-InterVac), University of Saskatchewan, Saskatoon, Saskatchewan Canada

## Abstract

Approximately 40% of HIV-1 infections occur in the female genital tract (FGT), primarily through heterosexual transmission. FGT factors determining outcome of HIV-1 exposure are incompletely understood, limiting prevention strategies. Here, humanized NOD-Rag1^−/−^ γc^−/−^ mice differentially reconstituted with human CD34+ -enriched hematopoietic stem cells (Hu-mice), were used to assess target cell frequency and viral inoculation dose as determinants of HIV-1 infection following intravaginal (IVAG) challenge. Results revealed a significant correlation between HIV-1 susceptibility and hCD45+ target cells in the blood, which correlated with presence of target cells in the FGT, in the absence of local inflammation. HIV-1 plasma load was associated with viral dose at inoculation and frequency of target cells. Events following IVAG HIV-1 infection; viral dissemination and CD4 depletion, were not affected by these parameters. Following IVAG inoculation, HIV-1 titres peaked, then declined in vaginal lavage while plasma showed a reciprocal pattern. The greatest frequency of HIV-1-infected (p24+) cells were found one week post-infection in the FGT versus blood and spleen, suggesting local viral amplification. Five weeks post-infection, HIV-1 disseminated into systemic tissues, in a dose-dependent manner, followed by depletion of hCD45+ CD3+ CD4+ cells. Results indicate target cell frequency in the Hu-mouse FGT is a key determinant of HIV-1 infection, which might provide a useful target for prophylaxis in women.

## Introduction

Human immunodeficiency virus (HIV-1) remains a major global and public health challenge^1,2^. Greater than 36.9 M individuals live with HIV world-wide, with more than 2 M new infections occurring each year^3^. Notably, 80% of infections arise as a result of vaginal, penile, or rectal exposure; thus HIV-1 is predominantly a sexually transmitted mucosal infection^4–7^. Approximately 40% of infections occur as a result of vaginal transmission^3,5^, and women now constitute greater than half of the global population of HIV positive individuals. Consequently, understanding susceptibility, transmission, and acquisition of HIV-1 in the female genital tract (FGT) is an important step towards halting the spread of HIV-1.

Information regarding early transmission events in women is derived from a combination of epidemiological studies, mathematical modeling and experimental studies in non-human primate (NHP) and human tissue explants^8–10^. The current dogma from these studies is that during heterosexual transmission HIV-1 in the ejaculate breaches the cervical or vaginal epithelium via microtears, transcytosis, or by percolating through the peri-cellular space, gaining access primarily to CD4+ target cells located in the submucosa^8,9,11^. After crossing the epithelial barrier and infecting a small population of CD4+ target cells, a short period of local viral amplification is thought to be required prior to migration of the virus outside the FGT, at which point it can be detected in peripheral blood^8,12^. This eclipse phase has been proposed to provide a window of opportunity to halt infection progression prior to the systemic spread of virus^8,9,13^. At present it is unclear precisely which factors determine successful infection following viral exposure. A productive infection has been shown to be dependent on whether or not HIV-1 is able to access sufficient target cells^14,15^. Whether this requires a pre-existing population of target cells in the tissue, or if recruitment of CD4+ target cells following induction of host innate immunity, pro-inflammatory cytokines, and inflammation at the site of transmission is necessary for successful infection, is not clear. The recruitment of target cells is believed to facilitate local viral amplification and dissemination into local lymph nodes and eventually the systemic circulation^8,9,13^. Studies have shown that within three to five weeks following infection HIV-1 establishes its characteristic reservoirs in the tissues (gut associated lymphoid tissue (GALT), spleen, liver, lung, brain, lymph nodes), indicating a productive systemic infection has occurred^8^. However, much of this current paradigm has been inferred from simian immunodeficiency virus (SIV) infections in macaques^8,9,15^. A recent study used a humanized mouse model to demonstrate that preventing leukocytes from responding to cytokines and chemokines halted viral escape from the FGT, and that blocking lymphocyte egress from lymph nodes prevented plasma viremia and systemic infection^13^. However, additional studies are needed to directly examine the determinants of HIV-1 infection during early infection.

The frequency of target cells at the site of mucosal transmission has been implicated as an important factor in determining the outcome of HIV-1 exposure, based on studies showing that elevated genital inflammation enhances the abundance of CD4+ HIV-1 target cells and is associated with increased risk of HIV infection^16–20^. Inflammation has also been shown to be integral for productive SIV infection following vaginal exposure in NHPs^21^. Moreover, sexually transmitted infections (STIs) increase inflammation in the genital tract, which correlates with increased risk of HIV-1 acquisition likely due to the enhanced recruitment of target cells^22^. However, in all of these studies the increase in target cells was accompanied by inflammation in the tissue. Very few, if any, studies have correlated target cell frequency with the outcome of HIV exposure, in the absence of inflammation. In addition to target cell frequency, viral dose is a clinically relevant factor to consider, as studies have shown that higher concentrations of HIV-1 RNA in serum and genital secretions (endocervical swab and semen) correlate with heterosexual transmission^23,24^. However, at present, the interaction between these key determinants (target cell frequency and viral dose) has not been described.

Here, using a heterosexual model of HIV-1 infection in humanized mice, we were able to systematically evaluate the individual effect of target cell frequency and viral dose, as well as their interactions following intravaginal (IVAG) challenge. We examined the frequency of human CD45+ target cells and viral inoculum dose as key determinants of the outcome of HIV-1 exposure. We also showed that following IVAG inoculation with HIV-1 in humanized non-obese diabetic NOD-Rag1^−/−^ γc^−/−^ (NRG) mice reconstituted with human CD34+ enriched hematopoietic stem cells (Hu-mice), viral replication occurs locally, followed by the systemic dissemination and depletion of CD4+ target cells. Our results demonstrate that the frequency of target cells supersedes viral dose at inoculation as the key determinant of successful HIV-1 infection in Hu-mice, even in the absence of local inflammation, and that the viral dose at inoculation determines subsequent viral titre in the plasma.

## Results

### Susceptibility to HIV-1 infection is associated with greater proportion of human CD45+ cells in the peripheral blood

Although, the frequency of HIV-1 target cells at the site of mucosal transmission has been implicated as an important factor, studies demonstrating the link between the frequency of target cells and risk of HIV infection are correlative. In most cases, genital inflammation was seen to enhance the abundance of CD4+ HIV-1 target cells and was associated with increased risk of HIV infection^16–20^. During our preliminary experiments establishing our Hu-mouse model of IVAG HIV-1 infection we, like others^25^, observed that the frequency of circulating human CD45+ target cells in the blood appeared to be correlated with whether or not Hu-mice would become infected following IVAG HIV-1 exposure. We therefore decided to exploit the Hu-mouse model, where each mouse is reconstituted to varying degrees with human immune cells, to assess whether the proportion of hCD45+ cells in peripheral blood is a direct determinant of IVAG infection.

**Figure 1:**
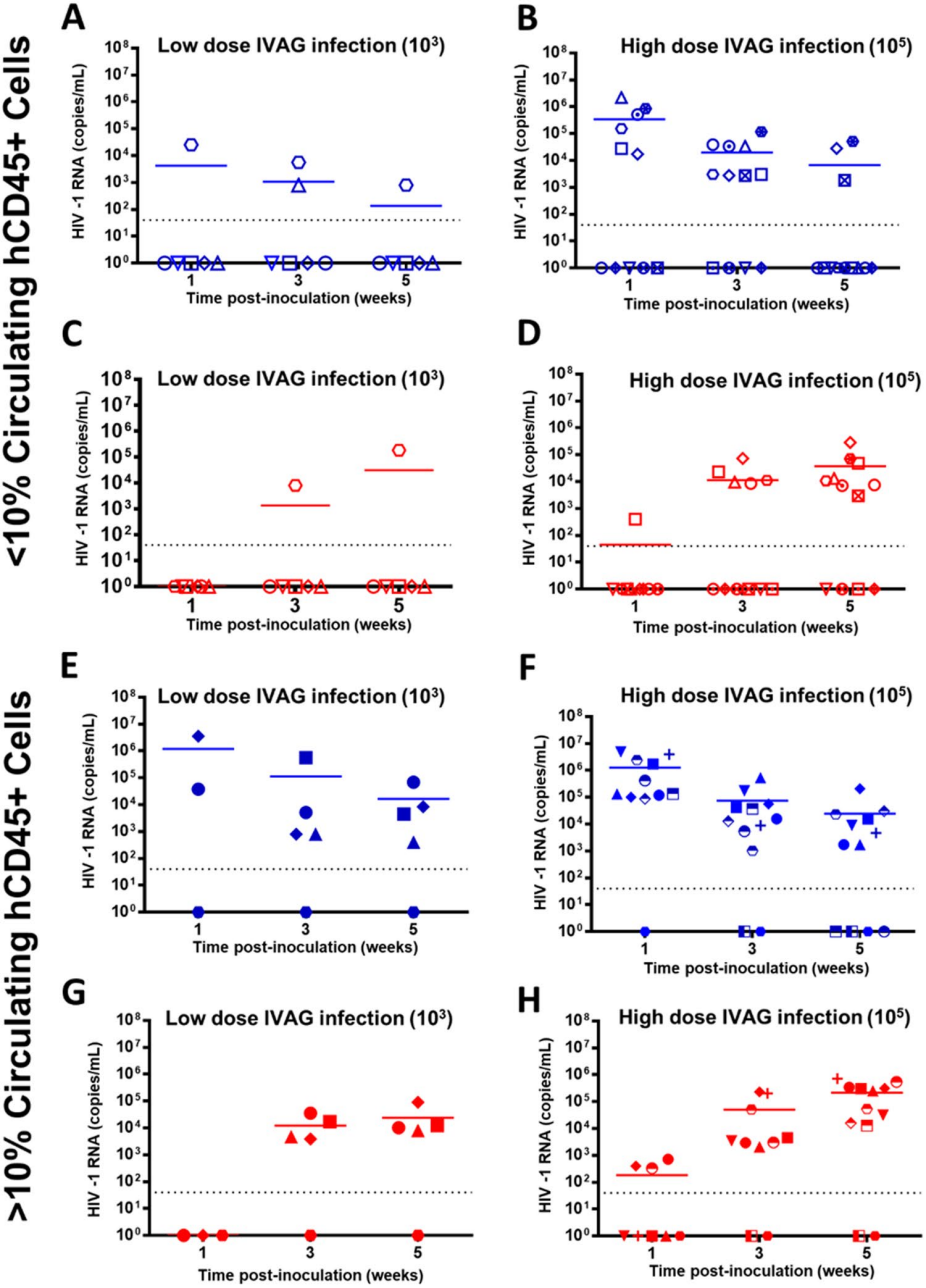
Greater than 10% human CD45+ cells in the peripheral blood is associated with higher susceptibility to HIV-1 transmission. Hu-mice with <10% circulating hCD45+ cells (**A**–**D**) and >10% circulating hCD45+ cells (**E–H**), identified by flow cytometry, were challenged IVAG with low (10^3^) (**A**, **C**, **E**, **G**) or high (10^5^ TCID_50_ (NL4.3-BAL)) (**B**, **D**, **F**, **H**) dose HIV-1. Paired viral titres (RNA copies/mL) were quantified in the vaginal lavage (blue) and plasma (red) of Hu-mice by clinical real-time RT-PCR. Infection did not occur in Hu-mice with <10% hCD45+ cells in the peripheral blood when challenged with low dose HIV-1 (**A**, **C** – 1/6 infected) while those challenged at high dose were more likely to be infected (**B**, **D** – 8/12 infected). Several Hu-mice with >10% hCD45+ cells in the peripheral blood became infected following IVAG challenge with both low (**E**, **G** – 4/5 infected) and high dose (**F**, **H** – 10/12 infected) HIV-1. Each symbol represents an individual Hu-mouse and paired titres are denoted by identical symbols on the corresponding graph (ie. **A** and **C**, **B** and **D**, **E** and **G**, **F** and **H**). Data was pooled from >3 independent experiments. One experiment did not include vaginal and plasma titres at week 1, thus some values for week 1 titres were omitted. IVAG: intravaginal.

Hu-mice reconstituted to varying levels of hCD45 (0.29–62.2%) were inoculated IVAG with either low dose (NL4.3-BAL 10^3^ TCID_50_, N = 17) or high dose HIV-1 (10^5^ TCID_50_, N = 17), and infection following viral challenge was determined by a positive HIV-1 viral titre in the plasma by 5 weeks post-viral exposure (Fig. 1). HIV-1 in the vaginal lavage and plasma were monitored over 5 weeks. Hu-mice were divided by reconstitution (< or >10% circulating hCD45), and the infection rate in those with <10% hCD45 was quite low (1/6), (Fig. 1A–D, Table 1) while those challenged at high dose were more likely to be infected (8/12). The infection rate in Hu-mice with >10% hCD45 was similar between those challenged with low (4/5) and high (10/12) dose HIV-1 (Fig. 1E–H). To validate our experimentally chosen threshold of < or >10% hCD45, the hCD45 reconstitution values from all ongoing experiments (N = 76) were divided into quartiles, and 56% of infected mice were within the 50^th^ (5.6% hCD45) to 100^th^ (62.2% hCD45) percentile. Using generalized linear modelling, adjusting for viral dose, the upper quartile (>16% hCD45) was significantly associated with successful infection following IVAG challenge (P = 0.012). We therefore continued to use 10% hCD45 as our putative threshold for determining whether or not we would expect a Hu-mouse to become infected following IVAG inoculation with HIV-1.

**Table 1:** HIV-1 infection rate (plasma titre >40 copies/mL) in humanized mice with <10% and >10% circulating hCD45+ cells 5 weeks after intravaginal challenge with low (10^3^ TCID_50_ HIV-1) or high (10^5^ TCID_50_ HIV-1) dose of viral inoculum.

	Low Dose (10^3^) Challenge	High Dose (10^5^) Challenge
<10% Circulating hCD45+ Cells	1/6 16.7%	8/12 66.7%
>10% Circulating hCD45+ Cells	4/5 80.0%	10/12 83.3%

### The proportion of human CD45+ cells in the peripheral blood supersedes viral dose at inoculation as the key determinant of HIV-1 infection in humanized mice

Having found that the number of CD45+ target cells in the peripheral blood and viral dose appeared to affect HIV-1 susceptibility, we employed statistical modelling to establish the correlates of IVAG HIV-1 infection in Hu-mice. In order to determine which factor (quantity of target cells in the peripheral blood or viral dose) was most important in determining if infection will occur following IVAG exposure to HIV-1, the association between HIV-1 infection in Hu-mice following viral challenge and covariables was estimated in a univariate (unadjusted) and multivariate (adjusted) generalized linear model (Table 2). In the univariate analysis Hu-mice with >10% circulating hCD45 (OR 1.42, 95% CI: 1.14–1.77; P = 0.003), elevated CD45*CD3 (%CD45 multiplied by %CD3, estimates CD3+ target cells as a percentage of lymphocyte population) (OR 1.02, 95% CI: 1.00–1.04; P = 0.023), or challenged with high viral dose (10^5^) (OR 1.36, 95% CI: 1.09–1.70; P = 0.007) were more likely to become infected following IVAG challenge. After adjusting for viral dose and CD45, Hu-mice with >10% circulating hCD45 had significantly greater odds of becoming infected following IVAG challenge (aOR 3.32, 95% CI: 1.20–9.65; P = 0.023). High viral dose trended but did not reach significance (aOR 2.70, 95% CI: 0.97–7.79; P = 0.060). Thus, the frequency of human CD45+ target cells was identified as the key determinant of IVAG HIV-1 infection in Hu-mice.

**Table 2:** The association between HIV-1 infection in humanized mice following viral challenge and listed covariables was estimated in a univariate (unadjusted) and multivariate (adjusted) generalized linear model. P < 0.05 was considered significant.

Variable	Generalized Linear Model of Infection Status gives the probability that a Humanized Mouse is Infected with HIV-1 following Intra-vaginal Challenge, based on the Variables Listed					
	Unadjusted OR (95% CI)	P	Adjusted OR (95% CI)^$^	P	Adjusted OR (95% CI)^#^	P
**Infectious Status (N = 76)**						
CD45 (>10%)	1.42 (1.14–1.77)	***0***.***003***	3.32 (1.20–9.65)	***0***.***023***	3.32 (1.20–9.65)	***0***.***023***
CD3 (% of CD45)	1.00 (0.99–1.01)	0.95				
CD45* CD3 (%)	1.02 (1.00–1.04)	***0***.***023***	1.09 (0. 9–1.23)	0.12		
Viral Dose (High)	1.36 (1.09–1.70)	***0***.***007***			2.70 (0.97–7.79)	0.060

^$^Adjusted by Dose.

^#^Adjusted by Dose and CD45.

### Tissue distribution of target cells is enhanced in humanized mice with greater than 10% human CD45+ cells in their peripheral circulation and is not due to local inflammation

**Figure 2:**
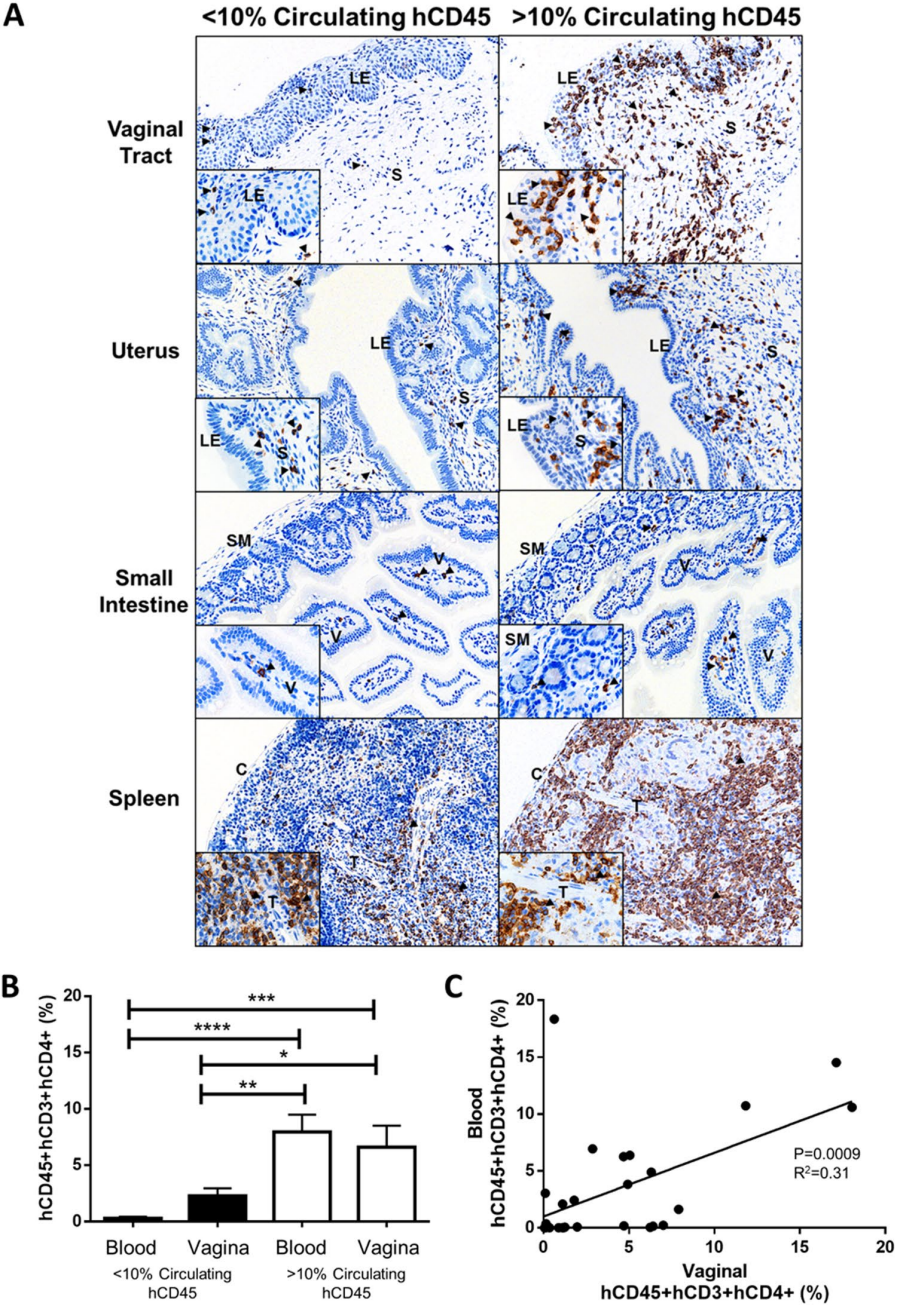
Tissue distribution of target cells is enhanced in humanized mice with greater than 10% human CD45+ cells in their peripheral circulation. (**A**) Vaginal tract, uterus, small intestine, and spleen from uninfected Hu-mice. Tissue sections from Hu-mice with < and > than 10% hCD45+ cells in the peripheral circulation were stained and compared for hCD3+ cells using AEC as a chromogen (brown colour) (**A**). and hCD4+ cells (%) were enumerated by flow cytometry using AEC as a chromogen (brown colour) (**B**) Immunoreactivity was observed in all tissues, however Hu-mice with >10% circulating hCD45 a greater abundance of target cells in their tissues (right vs. left panels). Within the vaginal tract, hCD3+ cells were localized within the luminal epithelium and stroma, while in the uterus, hCD3+ cells were mainly observed in the stroma. The small intestine had hCD3+ cells within the lamina propria of the intestinal villi, and the distribution of hCD3+ splenic T-cells was atypical, with T-cells dispersed throughout the spleen, particularly in the medulla. (**C**) The proportion of hCD45+ hCD3+ hCD4+ cells in the vaginal mucosa and peripheral blood were quantified by flow cytometry as a percentage of the total lymphocyte population. Uninfected Hu-mice with >10% circulating hCD45 (N = 11) had a significantly greater proportion of hCD45+ hCD3+ hCD4+ target cells in the vaginal mucosa, than Hu-mice with <10% circulating hCD45 (6.6 ± 1.9 vs. 2.3 ± 0.7%; P = 0.02; N = 18, ANOVA). (**C**) A significant correlation between hCD45+ hCD3+ hCD4+ cells in the blood and vaginal mucosa was observed (R^2^ = 0.31; P = 0.0009; N = 29) in control (never challenged) Hu-mice. Greater reconstitution of human target cells in Hu-mouse tissues is correlated with hCD45 reconstitution in peripheral blood. C: cortex, LE: luminal epithelium, M: medulla, S: stroma, SM: smooth muscle, T: trabecula, V: villus. Original magnification was (**A**) 100X, inset 400X, (**B**) 400X. (*P < 0.05, **P < 0.01, ***P < 0.001, ****P < 0.0001).

As the previous experiments established that 10% circulating hCD45+ reconstitution was a useful threshold for determining whether or not HIV-1 infection was likely to occur following IVAG viral exposure, we next determined whether hCD45+ reconstitution in the blood was related to tissue target cell reconstitution in the vaginal tract and other tissues including the small intestine and spleen, which are major sites for viral replication post-dissemination. Therefore, we examined hCD3+ (Fig. 2A) T-cell localization in the vaginal tract, uterus, small intestine, and spleen from uninfected (control) Hu-mice by immunohistochemistry. Within the vaginal tract, hCD3+ cells were localized within the luminal epithelium and stroma, while in the uterus, hCD3+ cells were mainly observed in the stroma. The small intestine had hCD3+ cells within the lamina propria of the intestinal villi, and the distribution of hCD3+ splenic T-cells was atypical, with T-cells dispersed throughout the spleen, particularly in the medulla. To directly assess tissue target cells, flow cytometric analysis was done to determine the frequency of CD4+ T-cells in peripheral blood and vaginal tissue of the same mice. Uninfected Hu-mice with >10% circulating hCD45 (N = 11) had a significantly greater proportion of hCD45+ hCD3+ hCD4+ cells in the vaginal mucosa (as a percentage of the total lymphocyte population, quantified by flow cytometry), than Hu-mice with <10% circulating hCD45 (6.6 ± 1.9 vs. 2.3 ± 0.7%; P = 0.02; N = 18; Fig. 2B). Further, a significant correlation between hCD45+ hCD3+ hCD4+ cells in the blood and vaginal mucosa was observed (R^2^ = 0.31; P = 0.0009; N = 29; Fig. 2C) in these control, uninfected Hu-mice. Thus, greater reconstitution of human target cells (hCD3+ and hCD45+ hCD3+ hCD4+) in tissues was correlated with greater hCD45 reconstitution in peripheral blood.

To rule out that increased target cells were not a result of local vaginal inflammation due to graft versus host disease (GVHD) induced by reconstitution with human immune cells, we quantified 13 human cytokines in the vaginal mucosa of uninfected Hu-mice with < and >10% circulating hCD45 (N = 6/group) (Supplementary Figure S1). No significant differences were observed for any of the cytokines quantified including hGM-CSF (P = 0.51), hIFN-γ (P = 0.33), hIL-1β (P = 0.86), hIL-4 (P = 0.71), hIL-6 (P = 0.18), hIL-8 (P = 0.16), hIL-10 (P = 0.39), hIL-12 (p70) (P = 0.21), hMCP-1 (P = 0.16), hTNF-α (P = 0.31), hIL-13 (P = 0.29), and hIL-5 (P = 0.99). Human IL-2 was below the assay limit of detection, and could not be quantified in our samples. We also quantified 10 murine cytokines in the vaginal mucosa of the same mice to determine if the proportion of human immune cells affected murine cytokines (Supplementary Figure S2). No significant differences were observed for any of the cytokines quantified including mGM-CSF (P = 0.50), mIL-1β (P = 0.91), mIL-2 (P = 0.42), mIL-6 (P = 0.18), mIL-10 (P = 0.11), mIL-12 (p70) (P = 0.89), mMCP-1 (P = 0.26), and mTNF-α (P = 0.10). Murine IFN-γ, and IL-4 were below the assay limit of detection, and could not be quantified in our samples. Thus, neither human nor mouse inflammatory cytokines were significantly different between Hu-mice with < or >10% circulating hCD45, and the enhanced susceptibility to HIV-1 seen in Hu-mice with >10% hCD45 could not be due to local inflammation present in the FGT.

### HIV-1 titres in the plasma are dependent on the viral dose at inoculation

Since frequency of target cells was more important than viral dose in determining the odds of HIV-1 infection following IVAG viral exposure, we next sought to determine whether plasma viral loads following infection were affected by target cell frequency or viral inoculation dose. The relationship between the HIV-1 titre in the plasma (Table 3), and covariables was estimated in a multivariate linear mixed effects model with repeated measures. After adjusting for time (weeks post-infection), dose, and CD45+ cells, higher viral dose (10^5^) was positively associated with greater plasma HIV-1 titres (adjusted β 1.98, 95% CI: 0.19–3.78; P = 0.032) than the lower viral dose (10^3^). In the adjusted model, the blood titre was also positively associated with >10% circulating hCD45+ cells (adjusted β 2.56, 95% CI: 0.83–4.29; P = 0.004). Taken together, these results indicate that the viral dose at inoculation is a key determinant of subsequent viral titre in the plasma, and that there is a significant correlation between viral titre in the plasma and frequency of HIV-1 target cells.

**Table 3:** The association between the plasma HIV-1 titre (copies/mL) in humanized mice and listed covariables was estimated in a multivariate linear mixed effects model with repeated measures. P < 0.05 was considered significant.

Variable	Linear Mixed Effects Model gives the probability that the Blood Titre for HIV-1 following Intra-vaginal Challenge depends on the Independent Variables Listed					
	Adjusted Regression Coefficient β (95%CI)^^^	P	Adjusted Regression Coefficient β (95%CI)^$^	P	Adjusted Regression Coefficient β (905%CI)^#^	P
**Plasma Titre (N=161)** ^**§**^						
CD45 (>10%)	3.27 (1.62–4.92)	***<0***.***001***	1.98 (0.83–4.29)	***0***.***004***	2.56 (0.83–4.29)	***0***.***004***
CD3 (% of CD45)	0.001 (−0.04–0.04)	0.96				
CD45*CD3 (%)	0.21 (0.07–0.40)	***0***.***005***	0.144 (−0.005–0.29)	0.062	1.98 (0.19–3.78)	***0***.***032***
Viral Dose (High)	2.98 (1.22–4,73)	***<0***.***001***				

^§^Total number of plasma titres available from mice at week 1, 3, and/or 5 post-infection.

^Adjusted by Time.

^$^Adjusted by Time and Dose.

^#^Adjusted by Time, Dose, and CD45.

### HIV-1 viral burden declines in vaginal lavage and increases in plasma following intravaginal infection

After having demonstrated that the proportion of target cells was important in determining whether or not infection will occur following IVAG HIV-1 exposure we sought to explore the kinetics of HIV-1 infection in our Hu-mice. Hu-mice were challenged with 10^5^ TCID_50_ HIV-1 via the IVAG or intraperitoneal (IP) route, as a systemic control. Three weeks post-infection viral titres in plasma were quantified by clinical real-time qPCR (Supplementary Figure S3). Systemically infected Hu-mice (IP, N = 10) had significantly higher plasma viral titres than those infected IVAG (N = 17) (209,594 ± 81,302 copies/mL vs. 43,368 ± 8,069 copies/mL respectively; P = 0.02).

**Figure 3:**
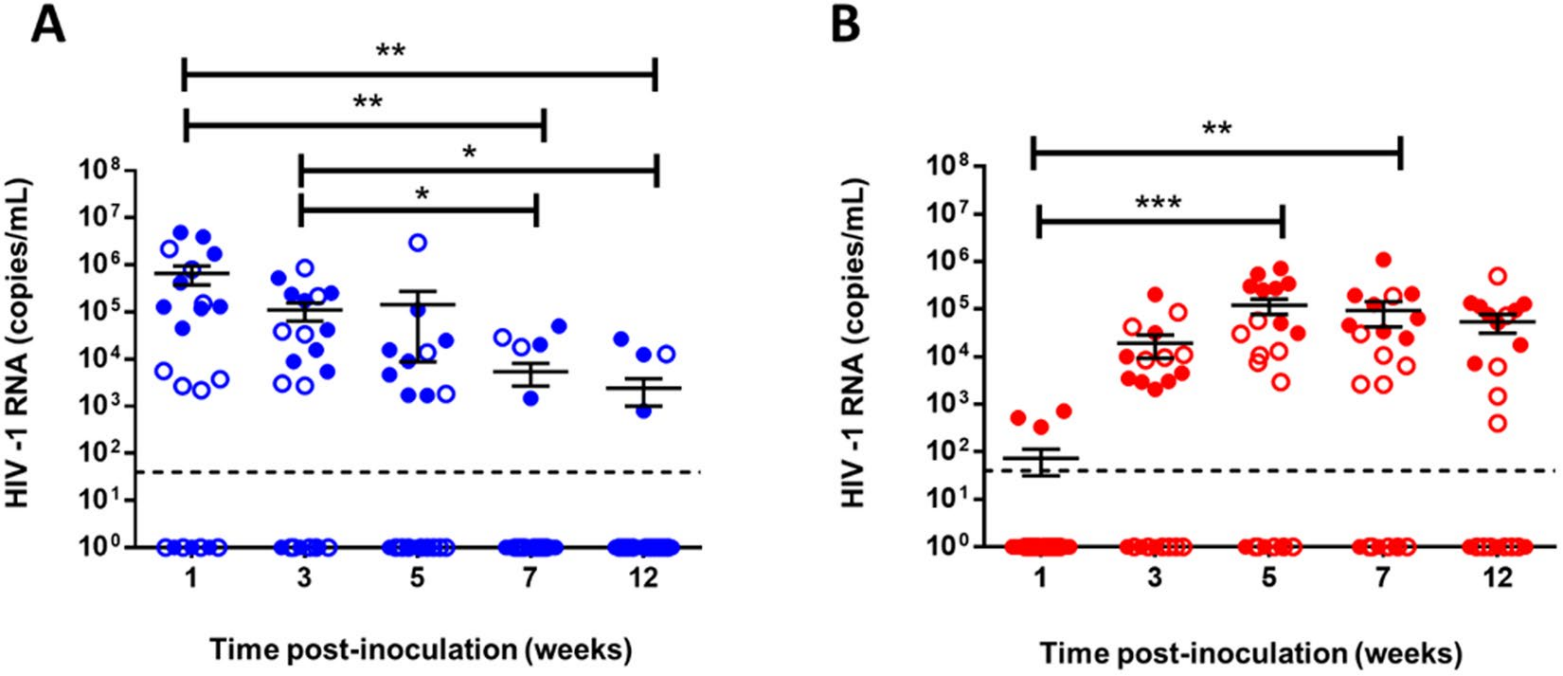
HIV-1 infection and viral burden in plasma and vaginal lavage. (**A**) HIV-1 RNA titers significantly decreased over time in the vaginal lavage (N = 22, ANOVA) while (**B**) plasma viral titers rose significantly in the 12 weeks following IVAG infection (N = 22, ANOVA). Hu-mice with <10% hCD45+ target cells in the peripheral circulation (N = 11/graph) are denoted by open dots, while those with >10% hCD45+ target cells in peripheral circulation are coloured. Data is shown as the mean ± Standard Error of the Mean (SEM). Each symbol represents an individual Hu-mouse and data was pooled from 2–3 independent experiments. Viral RNA was quantified by clinical real-time RT-PCR. Dashed line denotes PCR limit of detection. (*P < 0.05, **P < 0.01, ***P < 0.001).

Next we quantified viral titres in the vaginal lavage, and plasma by clinical real-time qPCR for 12 weeks following IVAG challenge with 10^5^ TCID_50_ HIV-1. Similar to previous studies^26^, we found that HIV-1 RNA in the vaginal lavage decreased post-infection, and reached significantly decreased levels by 7 weeks after IVAG infection, where they remained low to 12 weeks (N = 22) (Fig. 3A). This decline in titres occurred in Hu-mice with <10% circulating hCD45 (N = 11; Fig. 3A, open dots) (Supplementary Figure S4A) and Hu-mice with >10% circulating hCD45 (N = 11; Fig. 3A, blue dots) (Supplementary Figure S4C). Conversely, HIV-1 RNA in the plasma increased significantly over time and appeared to reach a viral set point by 5 weeks post-infection that was maintained to week 12 (N = 22) (Fig. 3B). This increase in titres was observed in Hu-mice with <10% circulating hCD45 (N = 11; Fig. 3B, open dots) (Supplementary Figure S4B) and Hu-mice with >10% circulating hCD45 (N = 11; Fig. 3B, red dots) (Supplementary Figure S4D). These results indicate that once infection is initiated, the infection kinetics are similar, regardless of level of reconstitution.

### Greater frequency of human CD3+ target cells expressing p24-antigen are detected locally in the vaginal tract during early infection

**Figure 4:**
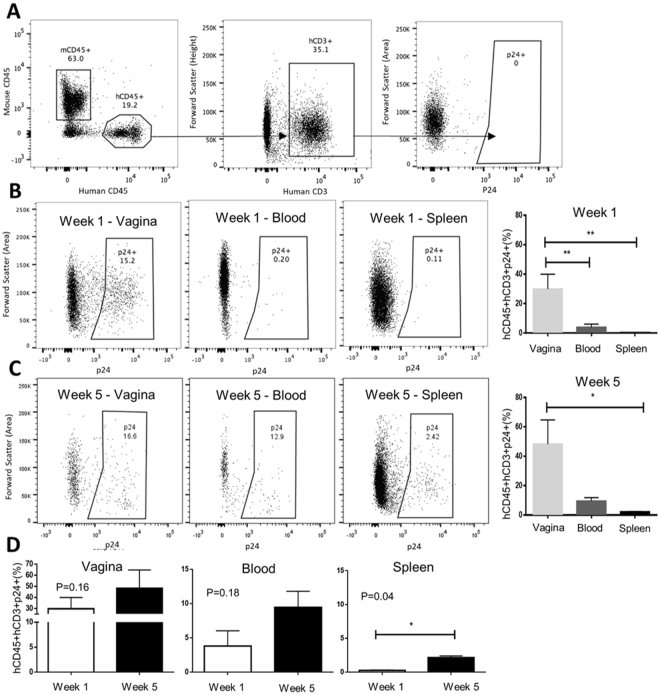
Expression of viral p24-antigen begins in the vaginal tract before spreading systemically. (**A**) A fluorescent minus one (FMO) negative control outlining the flow cytometry gating strategy used to assess p24 expression. Representative dot plots demonstrating expression of p24 by flow cytometry in hCD45+ hCD3+ cells isolated from the Hu-mouse vaginal tract, blood, and spleen at 1 week (N = 5) (**B**) and 5 weeks (N = 3) (**C**) following intra-vaginal (IVAG) infection with 10^5^ TCID50 HIV-1 (NL4.3-BAL). At 1 week post-infection, there were more hCD45+ hCD3+ p24+ cells in the vaginal tract than peripheral blood or spleen (29.9 ± 10.1% vs. 3.82 ± 2.2% vs. 0.26 ± 0.06; P = 0.004; N = 5, ANOVA) and the difference was sustained between the vaginal tract and spleen at 5 weeks (48.2 ± 16.6% vs. 2.2 ± 0.25%; P = 0.03; N = 3, ANOVA). (**D**) The percentage of hCD45+ hCD3+ p24+ cells in the vaginal mucosa and blood remained stable at 5 weeks while those in the spleen increased in frequency over time (0.26 ± 0.06% vs. 2.15 ± 0.25%; N = 5, 3; P = 0.04, t-test). (*P < 0.05, **P < 0.01).

Since the kinetics of viral infection was not affected by target cell reconstitution, we next examined viral dissemination from the initial site of infection (vaginal tract) into the systemic compartment (blood and spleen) at 1 week and 5 weeks following IVAG infection with 10^5^ TCID_50_ HIV-1, in Hu-mice with varying levels of human reconstitution (< and >10% hCD45). Infected human hCD45+ hCD3+ p24+ leukocytes were enumerated by flow cytometry following the fluorescent minus one (FMO) gating strategy outlined in Fig. 4A. Human CD3 was chosen for gating p24+ cells because hCD4 expression on the surface of HIV-1 infected cells is known to be downregulated^27–29^. At 1 week post-infection (Fig. 4B), there was a greater frequency of hCD45+ hCD3+ p24+ cells in the vaginal tract than peripheral blood or spleen (29.9 ± 10.1% vs. 3.82 ± 2.2% vs. 0.26 ± 0.06; P = 0.004; N = 5) and the difference was sustained between the vaginal tract and spleen at 5 weeks (48.2 ± 16.6% vs. 2.2 ± 0.25%; P = 0.03; N = 3) (Fig. 4C). The percentage of hCD45+ hCD3+ p24+ cells in the vaginal mucosa (P = 0.16) and blood (P = 0.18) remained stable but trended towards an increase over time while those in the spleen increased in frequency significantly between weeks 1 and 5 (0.26 ± 0.06% vs. 2.15 ± 0.25%; P = 0.04; N = 5, 3) (Fig. 4D), suggesting dissemination of HIV-1 from the mucosal site of infection to the systemic circulation and tissues thereafter. These results indicate that regardless of reconstitution level, once HIV-1 infection has occurred, early viral replication primarily occurs locally in the vaginal tract prior to disseminating into the peripheral blood and spleen.

### Rate and extent of HIV-1 dissemination depends on initial viral dose at challenge

**Figure 5:**
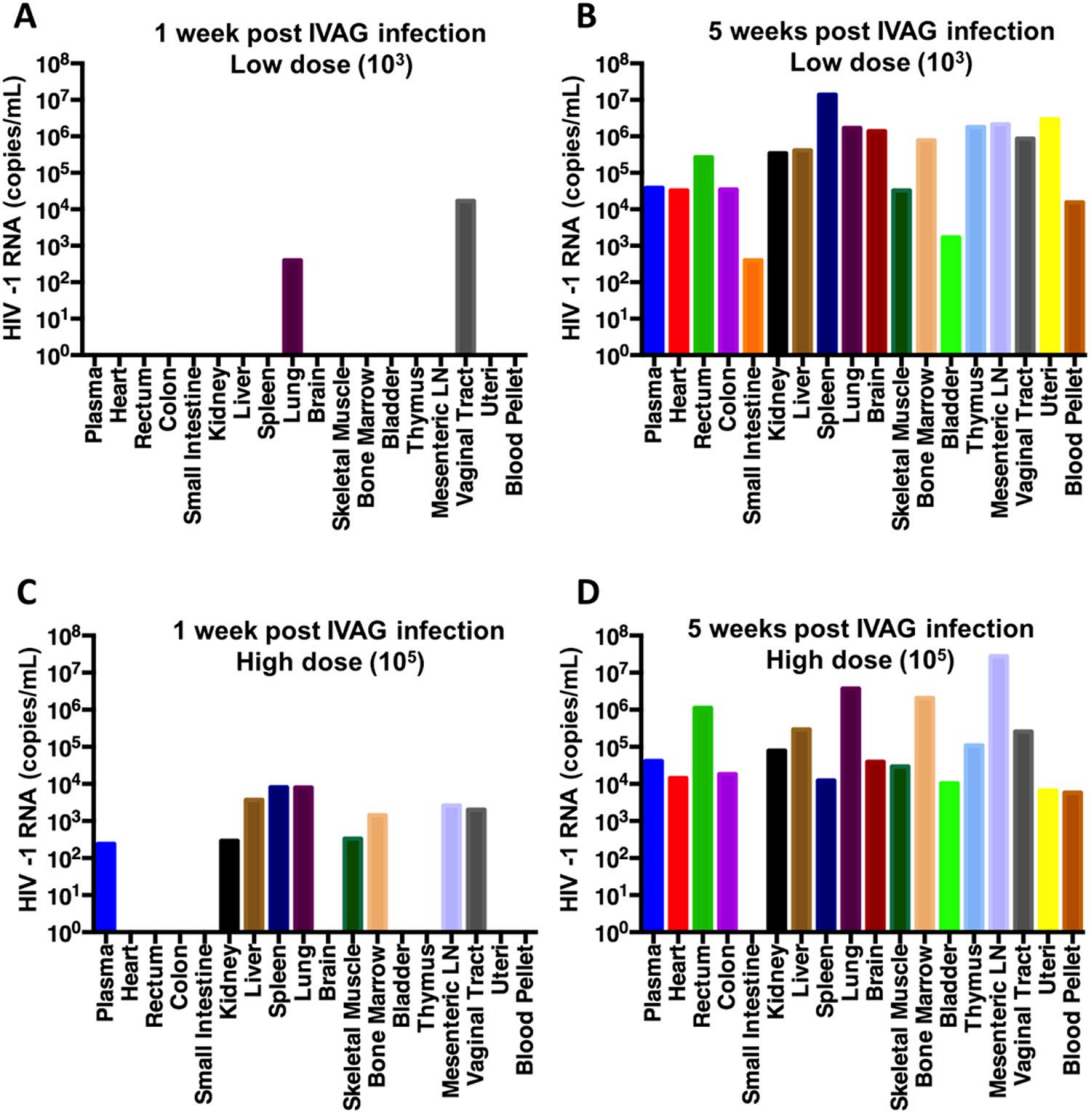
Extent of HIV-1 tissue dissemination in humanized mice varies 1 week following intravaginal infection with high or low dose viral inoculum. The proportion of tissues positive for HIV-1 RNA (copies/mL) as detected by clinical real-time RT-PCR increased between 1 and 5 weeks post-infection, in Hu-mice challenged IVAG with either a low (10^3^ TCID_50_ HIV-1 (NL4.3-BAL)) (**A**, **B**) or high dose (10^5^ TCID_50_ HIV-1 (NL4.3-BAL)) (**C**, **D**) inoculum of HIV-1. The magnitude of viral RNA increased between 1 and 5 weeks post infection regardless of initial viral dose (**A** vs. **B**, and **C** vs. **D**). At 1 week post-infection, HIV-1 RNA could be detected in the lung and vaginal tract of a Hu-mouse infected with low dose HIV-1 (**A**), whereas 50% (9/18) of tissues were positive in a Hu-mouse infected with high dose HIV-1 (**C**). By 5 weeks post infection, Hu-mice infected with a low (**B**) and high dose (**D**) HIV-1 had comparable viral dissemination. Each graph represents tissues collected from a single infected Hu-mouse. Tissue dissemination in additional Hu-mice is described in Table 4. IVAG: intravaginal, LN: lymph node.

Since our results thus far demonstrated that HIV-1 titres in the plasma were dependent on viral dose at inoculation, and that hCD45+ hCD3+ p24+ cells were most frequent in the vaginal tract early in HIV- infection, we next examined the kinetics and extent of viral dissemination following IVAG infection. Hu-mice with varying levels of human reconstitution (< and >10% hCD45) were infected IVAG with either a low (10^3^) or high (10^5^) TCID_50_ dose of HIV-1 to determine the effect of viral dose on subsequent tissue dissemination of virus, at 1 and 5 weeks following IVAG infection. Clinical real-time qPCR for HIV-1 was performed on a multitude of organs, tissues, and body fluids collected from Hu-mice (Fig. 5; N = 1/dose and week). The results were further expanded to a greater number of mice using an in-house HIV-1 PCR (Table 4; N = 3/dose and week), which had been validated against clinical real-time qPCR as described in Methods. As expected, the amount of HIV-1 RNA increased between 1 and 5 weeks post-infection, regardless of the initial viral dose (Fig. 5A vs. B, and C vs. D) or reconstitution. Both clinical real-time qPCR and the in-house PCR showed viral dissemination was more extensive 1 week after infection in Hu-mice that had been infected with high dose inoculum as compared to those inoculated at low dose (48% vs. 39% tissues infected, N = 3 per group, Table 4). Thus the kinetics of dissemination support the hypothesis that local viral amplification occurs prior to systemic dissemination, and expands upon a recent report demonstrating this phenomenon in BLT Hu-mice^13^. Further, the dose of virus in the inoculum determines the rate of dissemination during early infection. By week 5, Hu-mice infected with high and low dose inoculum had comparable viral dissemination (Fig. 5B vs. D). Taken together, these results suggest that HIV-1 underwent a local viral replication phase in the vaginal tract prior to dissemination, and that the rate and extent of viral dissemination depended on the length of infection, as well as on the initial dose of HIV-1.

**Table 4:** Dissemination of HIV-1 in humanized mice following intravaginal infection.

Mouse #	Uninfected Controls				Low Dose (10^3^) 1 Week				High Dose (10^5^) 1 Week				Low Dose (10^3^) 5 Weeks				High Dose (10^5^) 5 Weeks			
	112-5	110-4	5-1	Total	152-1	129-1	125-5	Total	125-4	120-1	123-2	Total	4-2	4-5	118-2	Total	5-3	2-2	1-3	Total
Plasma	−	−	−	0/3	+	−	−	1/3	−	+	−	1/3	+	+	+	3/3	+	+	+	3/3
Vaginal Wash	Not collected				+	+	+	3/3	−	+	+	2/3	−	+	+	2/3	−	−	+	1/3
Heart	−	−	−	0/3	+	−	−	1/3	−	−	+	1/3	+	+	+	3/3	+	+	+	3/3
Vaginal Tract	−	−	−	0/3	+	+	+	3/3	+	+	−	2/3	+	+	+	3/3	+	+	+	3/3
Rectum	−	−	−	0/3	+	−	−	1/3	+	−	+	2/3	+	+	+	3/3	+	+	+	3/3
Colon	−	−	−	0/3	+	−	−	1/3	−	−	+	1/3	+	+	+	3/3	+	+	+	3/3
Small Intestine	−	−	−	0/3	−	−	−	0/3	−	−	−	0/3	+	+	+	3/3	−	−	−	0/3
Kidney	−	−	−	0/3	+	−	−	1/3	−	+	−	1/3	+	+	+	3/3	+	+	+	3/3
Liver	−	−	−	0/3	+	−	−	1/3	+	+	−	2/3	+	+	+	3/3	+	+	+	3/3
Spleen	−	−	−	0/3	+	−	−	1/3	+	+	−	2/3	+	+	+	3/3	+	+	+	3/3
Lung	−	−	−	0/3	+	−	+	2/3	+	+	+	3/3	+	+	+	3/3	+	+	+	3/3
Brain	−	−	−	0/3	−	−	−	0/3	−	−	−	0/3	+	+	+	3/3	+	+	+	3/3
Skeletal Muscle	−	−	−	0/3	+	−	−	1/3	−	+	+	2/3	+	+	+	3/3	+	+	+	3/3
Bone Marrow	−	−	−	0/3	+	−	−	1/3	−	+	+	2/3	+	+	+	3/3	+	+	+	3/3
Uteri	−	−	−	0/3	+	−	−	1/3	−	−	+	1/3	+	+	+	3/3	+	+	+	3/3
Bladder	−	−	−	0/3	+	−	−	1/3	−	−	+	1/3	+	+	+	3/3	+	+	+	3/3
Thymus	−	−	−	0/3	+	−	−	1/3	−	−	+	1/3	+	+	+	3/3	+	+	+	3/3
Mesenteric LN	−	−	−	0/3	+	−	−	1/3	−	+	+	2/3	+	+	+	3/3	−	+	+	2/3
Infected Tissues	0/17	0/17	0/17	0/51 0%	16/18	2/18	3/18	21/51 0%	5/18	10/18	8/18	26/54 48%	17/18	18/18	18/18	53/54 98%	15/18	16/18	17/18	48/54 89%

### Intravaginal HIV-1 infection in humanized mice leads to a decrease in circulating human leukocytes over time

Finally, we sought to demonstrate the hallmark decline in circulating CD4+ T-cells over time, which has been shown to follow HIV-1 infection in humans, NHPs, and other Hu-mouse models^26^. PBMCs isolated from IVAG infected Hu-mice (mix of < and >10% hCD45 reconstitution) were stained and analyzed for hCD45, hCD3, and hCD4 by flow cytometry (Supplementary Figure S5) prior to infection (week 0; approximately 12–14 weeks following reconstitution with human cells) and at 1 or 12 weeks post-infection. Uninfected Hu-mice (control) had stable proportions of circulating hCD45+ cells over 7 weeks (5.6. ± 1.6% vs. 13.1 ± 1.8%, N = 6; P = 0.06), and a significant increase in the proportion of hCD45+ hCD3+ hCD4+ cells over the same time frame (47.3 ± 3.8% vs. 64.7 ± 4.5%, N = 6; P = 0.03). One week following infection, the percentage of hCD45+ (13.8 ± 5.7% vs. 20.8 ± 3.9%, N = 5; P = 0.06) and hCD45+ hCD3+ hCD4+ (47.0 ± 6.5% vs. 42.8 ± 13.0%, N = 5; P = 0.99) cells remained stable in Hu-mice infected IVAG with 10^5^ TCID_50_ HIV-1. By 12 weeks after infection, a significant decrease in hCD45+ (16.5 ± 4.7% vs. 1.2 ± 0.9%, N = 6; P = 0.03) and hCD45+ hCD3+ hCD4+ cells (18.6 ± 5.1% vs. 0.3 ± 0.1%, N = 6; P = 0.03) was observed. These results correspond with the viral replication data, indicating that in the first week post-infection when viral replication is primarily occurring in the vaginal mucosa, the hCD45+ and hCD4+ levels are sustained in peripheral blood, but by week 12, once virus has disseminated systemically, the frequency of hCD45+ and hCD4+ cells is significantly declined.

## Discussion

In this study we have addressed whether target cells and viral dose are critical determinants of vaginal HIV-1 infection in humanized mice. Importantly, we identify that the viral load in the inoculum and frequency of target cells present in the FGT are important determinants of the outcome of HIV-1 exposure, even in the absence of local inflammation in the FGT. We also demonstrate that they are relevant determinants of subsequent viral dissemination and plasma viral load in a heterosexual model of HIV-1 transmission. Furthermore, we demonstrate that although the initial infection is dependent on target cell frequency, once infection has occurred, it recapitulates the salient features of the paradigm of HIV-1 infection that have been proposed to occur following heterosexual infection in women, including local viral amplification followed by systemic spread, decline in vaginal titres, increase in plasma titres and decline in circulating CD4+ target cells over time, regardless of reconstitution level.

The development of Hu-mice in the mid-2000s has contributed greatly to HIV research^30^. Unlike NHP which have to be infected with SIV or SHIV, Hu-mice mice can be infected with HIV-1, which allows for examination of the interactions between HIV-1 and human immune cells, rather than studying surrogate interactions between NHP cells and SIV or SHIV. Thus, Hu-mice are a valuable tool to study HIV infection^31–34^. A number of models have been generated, and are widely used to study HIV pathogenesis^35–37^, or as pre-clinical tools to evaluate HIV-1 prevention and treatment strategies^33,38^ including the efficacy of anti-retroviral therapy (ART) in preventing IVAG HIV-1 transmission, and systemic pre-exposure prophylaxis (PrEP) for prevention of mucosal (rectal) and intravenous transmission. However, despite a number of similarities with human HIV-1 infection, few studies have examined the early events of HIV-1 transmission and dissemination following IVAG HIV-1 exposure in Hu-mice^13,31,32,39,40^. In the present study, we used Hu-mice to determine which factor, frequency of target cells or initial viral dose, is most important in determining if an HIV infection will occur following IVAG exposure in Hu-mice. Due to the uniqueness of the Hu-mice model, each mouse is reconstituted with a different proportion of target cells, allowing us to determine the threshold of target cell reconstitution required for infection. A similar *in vivo* study would be unfeasible in NHP given the ethics and cost concerns. Additionally, the number of target cells would theoretically be similar between individual NHP in the absence of any stimulation. The Hu-mice model enabled us to assess the correlation between the outcome of HIV-1 exposure with target cell frequency in the absence of inflammation, which is frequently a confounder in clinical studies.

Given the unique features of Hu-mice described above, we were able to assess whether the frequency of HIV-1 target cells and viral dose were key determinants of IVAG infection in the Hu-mouse model and examine the interaction between the two. We observed that Hu-mice challenged IVAG with low dose HIV-1 inoculum were more likely to become infected if they had been reconstituted with >10% circulating hCD45+ cells. We also demonstrated that these Hu-mice had significantly more target cells (hCD45+ hCD3+ hCD4+) in the vaginal mucosa as compared to Hu-mice with <10% circulating hCD45 (Fig. 2B,C), which likely explains their enhanced susceptibility to IVAG HIV-1 infection. Other studies in BLT Hu-mice have also demonstrated the presence of HIV-1 target cells throughout the FGT^26^. In the present report, the 10% cut-off was chosen based on our preliminary experiments, showing that when Hu-mice were stratified into quartiles, regression modelling found that the top quartile (Hu-mice with >16% hCD45+ cells in circulation) was significantly associated with successful HIV-1 infection following IVAG exposure. Furthermore, 56% of infected mice were within the 50^th^ (5.6% hCD45) to 100^th^ (62.2% hCD45) percentile, we thus selected 10%, a reconstitution level between 5.6% and 16% hCD45, as the threshold to assess whether infection following HIV-1 exposure was positively associated with the frequency of target cells. Interestingly, we found when the virus concentration in the inoculum increased, the infection rate equalized, and the reconstitution of the Hu-mice (< or >10% hCD45+ cells) seemed less relevant. Since both CD45 reconstitution and viral inoculum dose had an effect on infection outcome, linear regression modelling was employed to determine which factor, reconstitution with hCD45+ cells or viral dose, was the most important determinant of IVAG infection in Hu-mice. Regression modelling revealed Hu-mice with a greater proportion of target cells (>10% hCD45) were 3.32 times more likely than those with less target cells (<10% hCD45) to become infected with HIV-1 following IVAG exposure, regardless of viral dose. In the model, viral dose trended but did not reach significance, suggesting it might be a secondary determinant of HIV-1 infection in Hu-mice. Subsequent modelling found that although target cells were more critical in determining successful infection, HIV-1 titres in plasma were dependent on both the viral dose at inoculation, and the proportion of target cells. The viral inoculation dose was also found to affect the extent of HIV-1 tissue dissemination, but only during early infection (week 1). Initially, viral spread was limited in Hu-mice infected at the low viral inoculation dose as compared to the high dose, however by 5 weeks post-infection viral spread was comparable regardless of initial concentration of the virus in the inoculum. At 5 weeks post-infection HIV-1 had spread to all tissues collected, however in the Hu-mice infected with high dose of virus (10^5^) the small intestine was noticeably negative for viral RNA. This suggests that by 5 weeks post-infection complete T cell depletion in the small intestine of Hu-mice has likely occurred. Taken together, results suggest that the number of target cells present in the FGT is a primary determinant of HIV-1 infection in Hu-mice and that viral load at exposure is a secondary determinant of viral titre and dissemination, in a model of heterosexual HIV-1 exposure.

In the present study, we demonstrated a correlation between the quantity of HIV-1 target cells and the odds of HIV-1 infection following IVAG viral exposure in Hu-mice. This work supports and expands upon Stoddart *et al*. 2011, where enhanced susceptibility to HIV-1 was observed in Hu-mice derived on the NOD-SCID-gamma (NSG) strain as compared to the NOD-SCID, which was attributed to enhanced peripheral blood reconstitution^25^. We also show that Hu-mice with a greater proportion of circulating target cells tended to have greater frequency of target cells in the vaginal mucosa, which likely enhances the risk of IVAG HIV-1 infection. This corroborates studies in women and NHPs where the frequency of target cells in the FGT have been implicated in determining the outcome of infection following heterosexual exposure to HIV-1 or SIV, respectively^16,17,21^. We also know that STIs increase inflammation in the FGT, and correlate with increased target cells and risk of HIV-1^22^. However, in almost all of these studies inflammation was the underlying condition which was correlated with increased target cells and risk of infection, making it difficult to discern the effect of changes in target cell numbers alone, as inflammatory conditions induce additional confounding factors within the tissue microenvironment including inflammatory cytokines and chemokines that may also contribute to enhanced viral susceptibility^41^. Our model enabled us to highlight the link between frequency of target cells and HIV-1 infection, in the absence of inflammation and other confounding inflammatory factors. We ruled out that increased reconstitution with human immune cells resulted in increased inflammation due to graft versus host disease (GVHD) by quantifying inflammatory cytokines in vaginal tissue, which did not show any correlation with degree of reconstitution (Supplementary Figures S1 and S2). Thus, our results showing that target cell frequency was a key determinant of HIV-1 infection in a model of heterosexual transmission implies that reducing target cells and inflammation in the FGT might be important foci for HIV-1 prevention strategies.

While our study found the proportion of target cells in the Hu-mouse FGT to be of greater importance than the viral load in determining the outcome of infection following IVAG exposure to HIV-1, viral load was found to be a key determinant of viral dissemination during early infection and subsequent plasma titre. The relevance of viral load to HIV infection has been shown by a number of studies where viral load in the semen was correlated with HIV transmission in women^42^. Moreover, transmission is much more likely and efficient during the acute phase of HIV-1 infection, when the seminal viral load is highest^23,24^. Additionally, HIV Treatment as Prevention (TasP) has been crucial in reducing transmission, because for the most part ART suppresses viral load to undetectable levels. However, even though the blood viral load may be undetectable, seminal HIV shedding during the early phase of ART occurs^43,44^. Sheth *et al*., described approximately half of men within 6 months of initiating ART intermittently shed HIV in the semen despite undetectable viral loads in the blood, which was independent of ART regimen, drug levels in the semen, or genital co-infections^43,44^. Therefore, during heterosexual exposure the FGT can encounter a range of viral loads in the semen, depending on whether men are in the acute phase of HIV-1 infection, have recently begun ART, or have been on effective ART for some time. In our study, viral dose verged (P = 0.06) on being significantly associated with successful HIV-1 infection following IVAG exposure in Hu-mice. Extrapolating from our results, we suggest the number of target cells in the FGT is likely a primary determinant of heterosexual transmission of HIV-1 in women, followed closely by seminal viral load, which might instead be a determinant of plasma viral load and dissemination during early infection.

One limitation of the present study was the use of a highly infectious, homogeneous molecular HIV clone during viral challenges in Hu-mice. In women, HIV is under constant selection pressure by the host immune system, and as such mutations in viral RNA occur during viral replication that confer evolutionary advantages and allow virions to escape immune pressure^45–47^. Although we employed a homogeneous molecular clone, we suspect that use of a diverse quasispecies of HIV, which is more representative of naturally occurring HIV infections, could potentially be an influencing factor. It would provide another layer of complexity and an additional determinant of infection in that a larger inoculum, with greater viral diversity, might be more likely to infect than a smaller inoculum, with less viral diversity. Additionally, a greater proportion of target cells combined with the viral heterogeneity present in quasispecies HIV might affect viral dissemination and viral load following infection. These would be clinically relevant and timely avenues to pursue in future studies.

Herein, we found the frequency of target cells in Hu-mice was a key determinant of infection following IVAG HIV-1 exposure, even in the absence of inflammation. In prior studies in women and NHP, increased susceptibility to HIV has been correlated with inflammation, mainly by examining pro-inflammatory cytokines, and target cells. Here, our data clearly demonstrate that an increased proportion of HIV target cells is sufficient in increasing susceptibility in the absence of enhanced pro-inflammatory cytokines. We also identified viral dose as a determinant of viral dissemination and plasma titre during early mucosal infection, and suggest that variability in immune reconstitution should be considered when conducting experiments in Hu-mice. Our study provides new information regarding HIV-1 susceptibility, and expands upon prior studies outlining viral kinetics, tissue target cells, and dissemination in Hu-mouse models of heterosexual transmission^13,26,31,39^. We strengthen the literature demonstrating that HIV-1 infection in Hu-mice recapitulates what is believed or known to occur following infection in women. Hu-mice can provide valuable information regarding early events following HIV-1 exposure in the female genital tract. One such corollary from the current study might be that conditions such as co-infections and inflammation, which increase target cells in the FGT, are key risk factors for increased HIV-1 susceptibility in women. Based on these results, we suggest that decreasing target cells in the FGT and viral load in the semen may be important primary and secondary goals of prevention strategies in women.

## Methods

### Study approval

All experimental protocols were approved by the Hamilton Integrated Research Ethics Board (HiREB) and McMaster University Animal Research Ethics Board (AREB) as per AUP# 14-09-40, in accordance with Canadian Council of Animal Care (CCAC) guidelines.

### Generation of humanized mice and immune reconstitution screening

A breeding pair of *NOD-Rag1* ^*tm1Mom*^ *Il2rg* ^*tm1Wjl*^ (NRG) mice was obtained from Jackson Laboratories (Bar Harbor, ME, USA) and bred in specific pathogen-free ultraclean rooms within the Central Animal Facility (McMaster University, Hamilton, ON, Canada). Hu-mice were generated as previously described^48^. Briefly, umbilical cord blood was obtained with parental consent from healthy newborns in the Department of Obstetrics and Gynecology at the McMaster University Medical Centre. The umbilical cord blood was processed using the RosetteSep Human Cord Blood Progenitor Enrichment Kit from Stem Cell Technologies (Vancouver, BC, Canada), to purify and enrich CD34^+^ cells. Purified CD34^+^ cells were cryopreserved until required. Four day old NRG pups were sub-lethally irradiated with a single-dose γ-ray (300 cGy) and then administered an intrahepatic injection with 1 × 10^6^ to 2 × 10^6^ CD34-enriched hematopoietic stem cells. The degree of human immune cell reconstitution in the NRG mice was evaluated by flow cytometry 90–120 days following the intrahepatic injection by quantifying the expression of human leukocyte markers, hCD45, hCD3, hCD4, as well as mouse CD45 in peripheral blood mononuclear cells (PBMCs) to determine the percent reconstitution in each Hu-mouse. Approximately 4-5 mice were reconstituted by a single cord blood donor, and throughout experiments mice were randomized based on their reconstitution (> or <10% circulating hCD45). Immune reconstitution did not appear to be dependent on donor (Supplementary Figure S6).

### Generation of NL4.3-Bal-Env HIV-1 for intravaginal infection

Viral preparations were generated by transient transfection of 293 T human embryonic kidney cells (HEK293T) using the calcium phosphate precipitation technique as described previously^49^. In brief, HEK293T cells were plated at a concentration of 4 × 10^6^ cells per 175 cm^2^ flask and transfected after 24 hours with 50 µg of the pNL4.3 Bal Env HIV-1 vector^50^. At 48 hours after transfection, cell supernatant containing viral particles was collected and concentrated 50 times by ultracentrifugation (90 min, 28000 g, 4 °C) before a final reconstitution in PBS. Multiplicity of infection (MOI) was determined by calculating the TCID_50_ using the Spearman-Karber method, by infecting TZM-Bl indicator cells with a serial dilution of viral stocks^51–55^.

### Intravaginal HIV-1 challenge

Female Hu-mice were estrous cycle staged by microscopically assessing vaginal cytology^56–59^. Hu-mice in the progesterone high/diestrus stage of the estrous cycle were anesthetized by IP injection of anesthetic (150 mg ketamine per kg/10 mg xylazine per mL). Mice were placed in a supine position, the vaginal tract was gently swabbed and an IVAG inoculation with a 25 µl dose of 10^3^ (low) to 10^5^ (high) TCID_50_/mL of NL4.3-Bal-Env HIV-1 was delivered, by gently inserting the viral inoculum using a pipette. Hu-mice remained in this position as they recovered from anaesthetic (~1 hour).

### Collection of vaginal lavage and plasma for quantification of HIV-1 titre

To track viral kinetics over time, vaginal lavage was collected by pipetting 2 × 30 μl of sterile phosphate buffered saline (PBS) (McMaster Media Stores, Hamilton, ON, Canada) in and out of the vaginal tract 3–4 times. Vaginal lavage was subsequently frozen at −80 °C until required. Peripheral blood was collected into plasma separator tubes (BD Biosciences, San Jose, CA, USA) by cheek or orbital bleeds. Plasma was isolated by centrifuging whole blood at 15 000 rotations per minute (RPM) for 8 minutes. Blood plasma was subsequently frozen at −80 °C until required. Frozen plasma and vaginal washes were sent in batches, on dry ice, to the Mt. Sinai Microbiology Laboratory (Toronto, ON, Canada) for clinical real-time PCR quantification of HIV-1 RNA using the Abbott RealTime HIV-1 m2000 (Abbott Laboratories, Illinois, USA). The sensitivity and specificity of this assay are 91.8% and 100% respectively^60^, and its lower limit of detection is 40 copies/mL.

### Isolation of peripheral blood mononuclear cells, vaginal cells, and splenocytes for flow cytometry

Whole blood was collected by cheek bleed (as above) or at experimental endpoint by cardiac puncture. Vaginal tracts were enzymatically digested with collagenase A 150 U/mL (Roche Diagnostics, Mississauga, ON, Canada) at 37 °C for 2 hours (two 1 hour incubations) as previously described^61^. Spleens were mechanically disrupted using the plunger of a 1 mL syringe (BD Biosciences, San Jose, CA, USA) and filtered through a 40 µm cell strainer (Thermo Fisher Scientific, Waltham, MA, USA). Erythrocytes were eliminated from splenocyte preparations with a 4 minute incubation in 2 mL of ACK lysis buffer (Thermo Fisher Scientific), and neutralized with PBS. PBMCs were recovered from whole blood after two rounds of ACK lysis buffer (2 mL, 4 mins; 500μL 1 min), neutralized by PBS. The resulting cell preparations (vagina, spleen, PBMC) were counted on a hemocytometer, resuspended to a final concentration of 1–3 × 10^6^/ml and stained for flow cytometry as below.

### Flow cytometry

Vaginal, splenic, and PBMCs were isolated as above and stained for 30 minutes with a cocktail of antibodies [hCD45 – V450, hCD4 – PerCP-Cy5.5, hCD8 – PE-Cy7 (BD Biosciences, San Jose, CA, USA); hCD3 – BV605 (Biolegend, San Diego, CA, USA); mCD45 – AF700 (eBioscience, San Diego, CA, USA)], following 20 minutes of Fc receptor blocking (eBioscience, San Diego, CA, USA) on ice. For intracellular staining (p24-antigen), cells were subsequently permeabilized and fixed with BD Cytofix/Cytoperm^TM^ Solution (BD Biosciences, San Jose, CA, USA) for 20 minutes, followed by a 30 minute incubation with the intracellular p24 antibody [p24 – PE (Beckman Coulter, Mississauga, ON, Canada)]. Within 24 hours of staining, data was acquired on the BD LSRII or BD LSR Fortessa flow cytometer (BD Biosciences, Canada). Data was analyzed and cell frequencies and phenotype were assessed using FlowJo® software (Version 10.0.8) (Treestar, Ashland, OR, USA).

### Quantification of HIV-1 RNA by real-time PCR in tissue homogenates

Tissues (heart, rectum, colon, small intestine, kidney, liver, spleen, lung, brain, skeletal muscle, bone marrow, bladder, thymus, mesenteric lymph node, vaginal tract, and uterus) were collected at experimental endpoint and mechanically homogenized in PBS, using metal beads and the Gold BulletBlender system (Next Advance, Averill Park, NY). Whole blood obtained by cardiac puncture was separated using plasma separator tubes (BD Biosciences, San Jose, CA, USA) into plasma and the cellular blood pellet, which was also homogenized in PBS. Homogenates were centrifuged at 8000 RPM for 5 minutes, and the supernatants were collected and stored at −80 °C until required. Tissue supernatants from 4 humanized mice were sent to the Mt. Sinai Microbiology Laboratory (Toronto, ON, Canada) for clinical real-time PCR quantification of HIV-1 RNA as above (Fig. 5). The presence or absence of HIV-1 was assessed in the remaining supernatants, plasma, and vaginal washes using an in-house TaqMan RT-PCR as follows (Table 4). Briefly, RNA was extracted from 50 µl of plasma, vaginal lavage, or homogenized tissue supernatant using the QIAamp MinElute Virus Spin Kit (QIAGEN, Toronto, ON, Canada) according to the manufacturer guidelines. Real Time RT-PCR was performed on the StepOne^TM^ Real Time PCR System (Thermo Fisher Scientific) using SensiFAST^TM^ Probe Hi-ROX One-Step Kit from Bioline (FroggaBio, Toronto, ON, Canada) containing 500ng RNA extract, 400 nM primers (HIV LTR Forward: 5′-GCCTCAATAAAGCTTGCCTTGA, Reverse 5′-GGCGCCACTGCTAGAGATTTT), and 100 nM reporter probe, previously published by Rouet *et al*. 2005^62^ (5′-AAGTAGTGTGTGCCCGTCTGTTRTKTGACT 5′ reporter 6-carboxyfluorescein and the 3′ quencher 6-carboxytetramethylrhodamine). Chronically infected H9 cell (NIH AIDS Reagent Program, Germantown, MD, USA) supernatants were used as a positive control, and HSV-2 infected Vero cells as a negative control. A series of samples were run on both the in house RT-PCR as well as at Mt. Sinai Hospital Microbiology Lab for quantitative clinical RT-PCR (as above), to validate and determine the sensitivity of the in house method. The threshold of the in house PCR was 15,000 copies/mL, any sample found to be below the in-house detection limit was sent to the Mt. Sinai Hospital Microbiology Lab for verification.

### Multiplex Cytokine Assays

A strip of vaginal tissue (1/4 of the vaginal tract split lengthwise) was collected from uninfected Hu-mice at endpoint and mechanically homogenized in PBS as above. Vaginal homogenates were centrifuged and cytokines were quantified in duplicate in the supernatants using the Human Cytokine Array Focused 13-plex (HDF13) and Mouse Cytokine Array/Chemokine Array 13-plex Secondary Panel (MD13) (Eve Technologies, Calgary, AB, Canada).

### Human CD3 immunohistochemistry

At endpoint, tissues were excised from uninfected Hu-mice, fixed in 10% formalin, processed, and embedded. Sections were cut at 4 µm, deparaffinized, and stained following a 5% H_2_O_2_ endogenous peroxidase block, antigen retrieval in citrate buffer, and blocking with 5% normal goat serum in TBS-T (20 mins). Sections were incubated with rabbit anti-human CD3 (RM-9107-S; Thermo Scientific; Waltham, Massachusetts, USA), diluted 1:100 in Ultra Clean Diluent (Fisher Scientific; Ottawa, ON) for 1 hour. Slides were rinsed in TBS-T before Dako’s Envision plus-rabbit (K4008; Dako Canada Inc; Burlington, ON) was added for 30 minutes. The slides were rinsed, and 3-Amino-9-ethylcarbazole (AEC) (Sigma-Aldrich; Oakville, ON) was employed as a chromogen (20 mins). Slides were counterstained with Mayer’s Hematoxylin (Sigma-Aldrich), 25 seconds. Sections were rinsed and coverslipped with glycerine gelatin. Images were visualized using an Olympus IX81 inverted microscope (Olympus, Richmond Hill, ON, Canada), and captured using the Infinity camera (Lumenera Corp., Ottawa, ON, Canada).

### Statistics

The majority of the data was analyzed and graphed using GraphPad Prism 6 (GraphPad Software, La Jolla, CA). Flow cytometry data was analyzed using FlowJo software (Treestar). Data were considered statistically significant if the P values obtained with a two-tailed *t*-test, analysis variance of the mean (ANOVA), or the equivalent non-parametric tests if the data was not normally distributed, and Spearman r correlation were <0.05. Significant differences are noted as ^*^P < 0.05, ^**^P < 0.01, or n.s. (not significant).

### Statistical modelling

To assess the relationship between HIV-1 susceptibility in humanized mice following viral challenge (Table 3), and between the HIV-1 titre in the and plasma (Table 4), with listed covariates (%CD45+ cells, %CD45+ CD3+ cells, CD45+ *CD3+ (%)), and viral dose) we performed univariate (unadjusted) and multivariate (adjusted) generalized linear models, and a multivariate linear mixed effects model with repeated measures using the glm and lmer functions in the stats and lme4 package in R version 3.2.3 (R Core Team, 2015). In the univariate model, the odds ratio (OR) and 95% confidence interval (CI) were calculated for each covariate. In the multivariate model, the adjusted odds ratios (aOR) and 95% CIs were estimated after adjusting for covariates. Viral titres underwent logarithmic transformation prior to analysis. In the multivariate model the adjusted regression coefficients (β) and 95% CIs were calculated. For all statistical models, a P < 0.05 was considered statistically significant.

### Data availability

The datasets generated during and/or analyzed during the current study are available from the corresponding author on reasonable request.

## Electronic supplementary material

Supplementary Information
