# Supplementary material for: Frequency of Human CD45+ Target Cells is a Key Determinant of Intravaginal HIV-1 Infection in Humanized Mice
Source: Sci Rep. 2017 Nov 10;7:15263. doi: 10.1038/s41598-017-15630-z (PMC5681573; doi:10.1038/s41598-017-15630-z)
Supplement: Supplementary file 1 — Supplementary Information [file 41598_2017_15630_MOESM1_ESM.pdf]

Supplementary Figures

Title: Frequency of Human CD45+ Target Cells is a Key Determinant of Intravaginal HIV-1 Infection in Humanized Mice

Authors: Philip V. Nguyen[1,2#], Jocelyn M. Wessels[1,2#], Kristen Mueller[1,2], Fatemeh Vahedi[1,2], Varun Anipindi[1,2], Chris P. Verschoor[2], Marianne Chew[1,2], Alexandre Deshiere[3], Uladzimir Karniychuk[1,2,$], Tony Mazzulli[4,5,6], Michel J. Tremblay[3], Ali A Ashkar[1,2], and Charu Kaushic[1,2*]

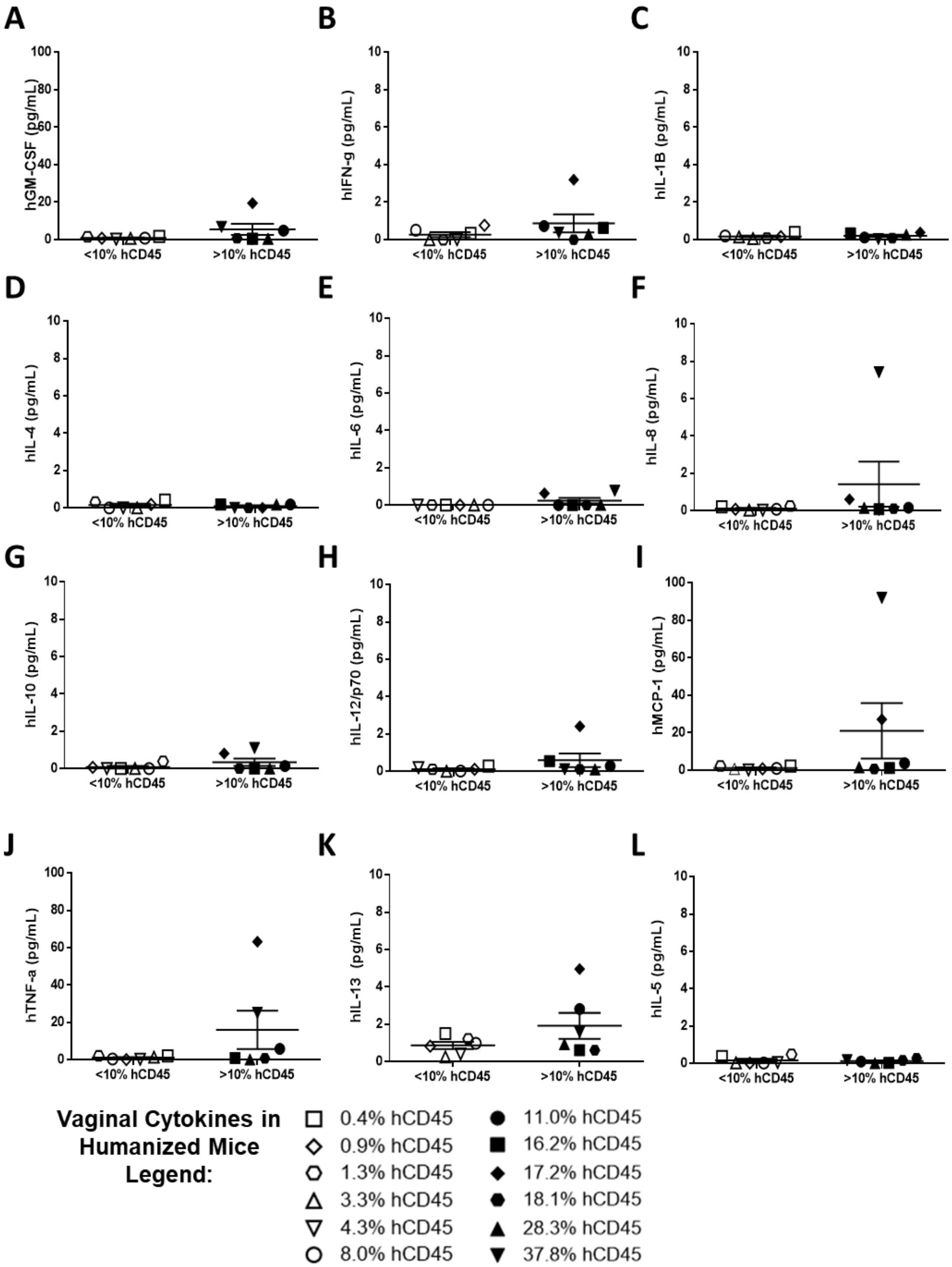

Supplementary Figure S1. Quantification of Human Cytokines in the Vaginal Mucosa of Humanized Mice. Human cytokines were quantified by multiplex cytokine array in the vaginal mucosa of uninfected Hu-mice with < and >10% circulating hCD45 (N=6/group). No significant differences were observed for any of the cytokines quantified including hGM-CSF (P=0.51) (A), hIFN-γ (P=0.33) (B), hIL-1β (P=0.86) (C), hIL-4 (P=0.71) (D), hIL-6 (P=0.18) (E), IL-8 (P=0.16) (F), hIL-10 (P=0.39) (G), hIL-12 (p70) (P=0.21) (H), hMCP-1 (P=0.16) (I), hTNF-α (P=0.31) (J), hIL-13 (P=0.29) (K), and hIL-5 (P=0.99) (L). Human IL-2 was below the assay limit of detection, and could not be quantified in these samples.

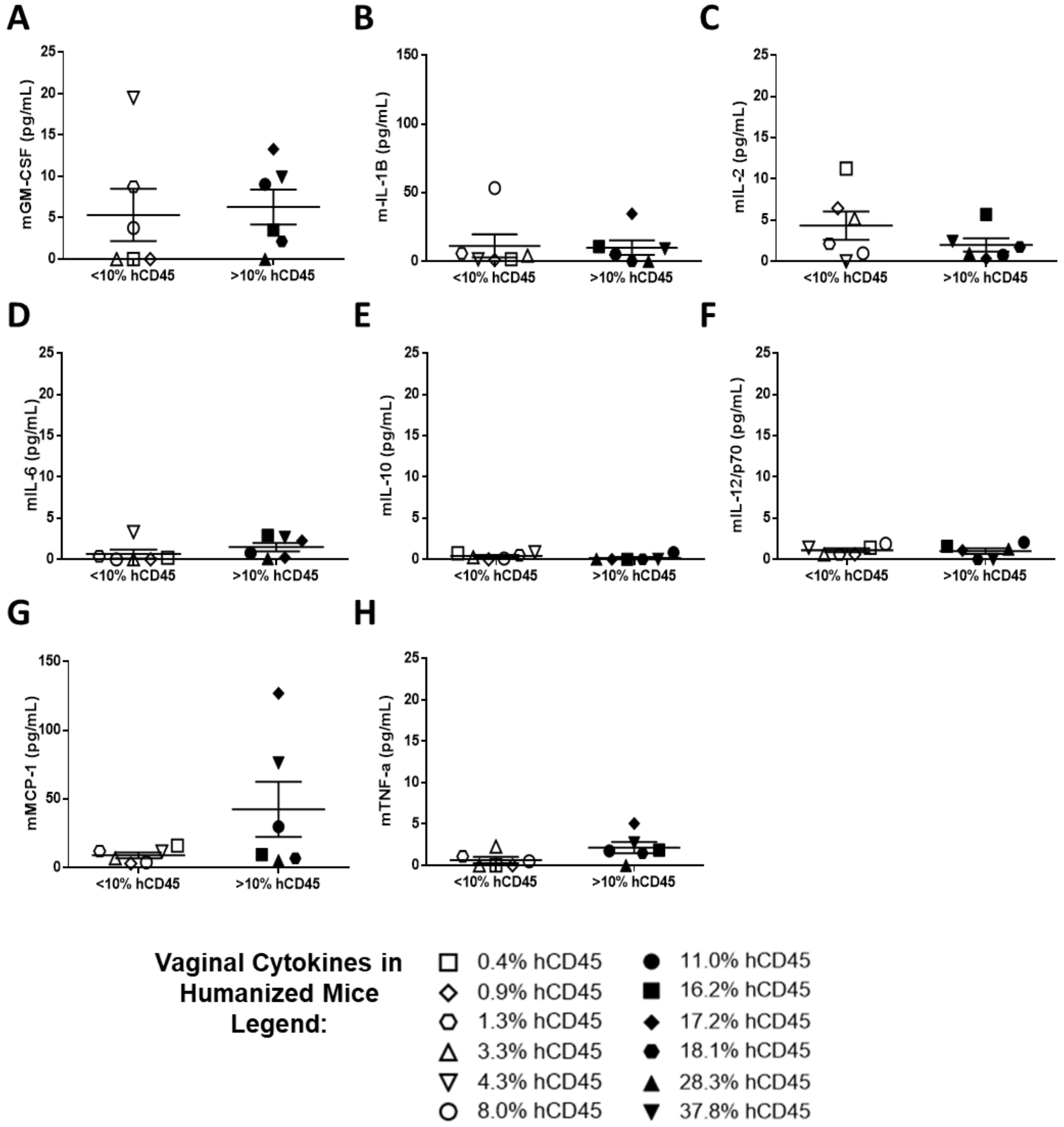

Supplementary Figure S2. Quantification of Murine Cytokines in the Vaginal Mucosa of Humanized Mice. Murine cytokines were quantified by multiplex cytokine array in the vaginal mucosa of the same uninfected Hu-mice (N=6/group) to determine if the proportion of human immune cells affected murine cytokines. No significant differences were observed for any of the cytokines quantified including mGM-CSF (P=0.50) (A), mIL-1β (P=0.91) (B), mIL-2 (P=0.42) (C), mIL-2 (P=0.18) (D), mIL-10 (P=0.11) (E), mIL-12 (p70) (P=0.89) (F), mMCP-1 (P=0.26) (G), and mTNF-α (P=0.10) (H). Murine IFN-γ, and IL-4 were below the assay limit of detection, and could not be quantified in these samples.

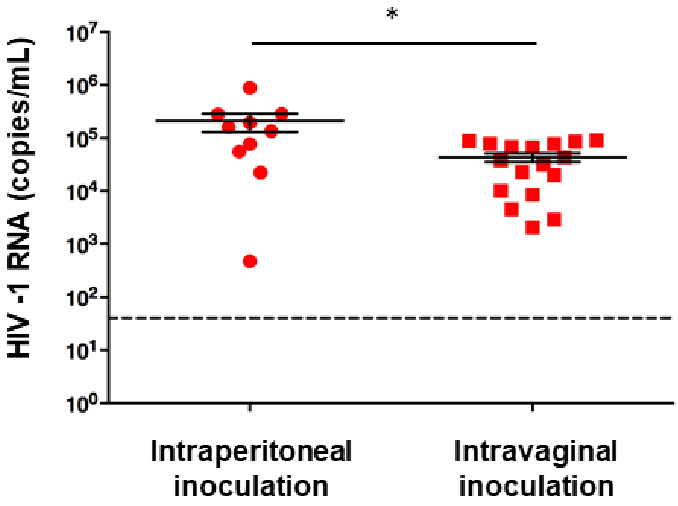

Supplementary Figure S3. Systemic versus IVAG infection. HIV-1 RNA (copies/mL) were quantified 3 weeks post-infection in the plasma of Hu-mice inoculated intraperitoneally (IP; N=10) or intravaginally (IVAG; N=17) with 10^5 TCID50 HIV-1 (NL4.3-BAL). Hu-mice infected by the IP route had significantly higher plasma viral titers than mice infected by the IVAG route (209,594±81,302 copies/mL vs. 43,368±8,069 copies/mL; P=0.0235, t-test) respectively. (*P<0.05).

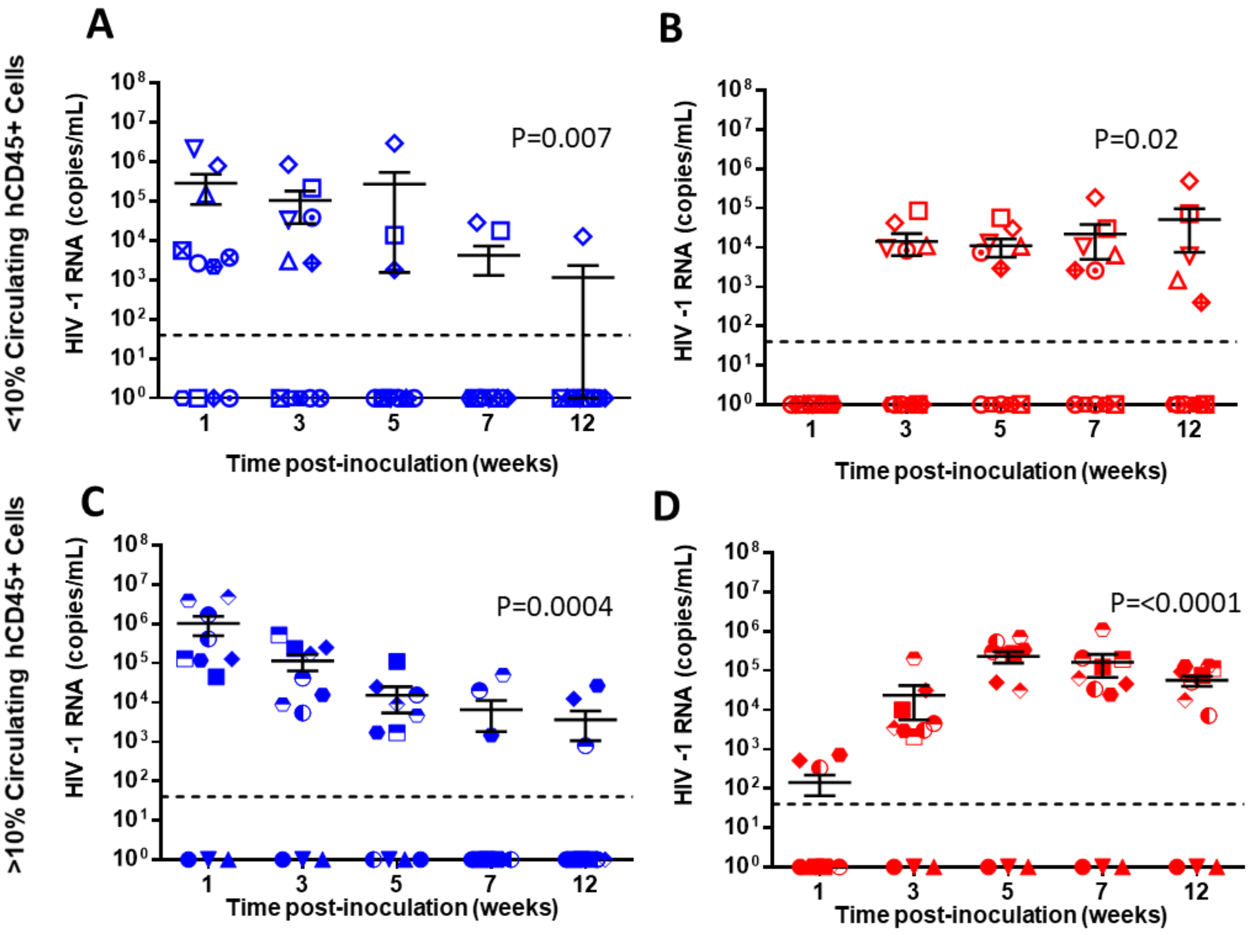

Supplementary Figure S4. HIV-1 infection and viral burden in plasma and vaginal lavage by reconstitution. HIV-1 RNA titers significantly decreased over time in the vaginal lavage of Hu-mice with <10% circulating hCD45 (N=11, ANOVA) (A) and rose significantly in the plasma in the 12 weeks following IVAG challenge in Hu-mice (N=11. ANOVA) (B). Vaginal (C) and plasma titres (D) followed a similar pattern in Hu-mice with >10% hCD45 (N=11, ANOVA). Each symbol represents an individual Hu-mouse and paired titres are denoted by identical symbols on the corresponding graph (ie. A and B, C and D). Data is shown as the mean ± Standard Error of the Mean (SEM). Data was pooled from 2-3 independent experiments. Viral RNA was quantified by clinical real-time RT-PCR. Dashed line denotes PCR limit of detection.

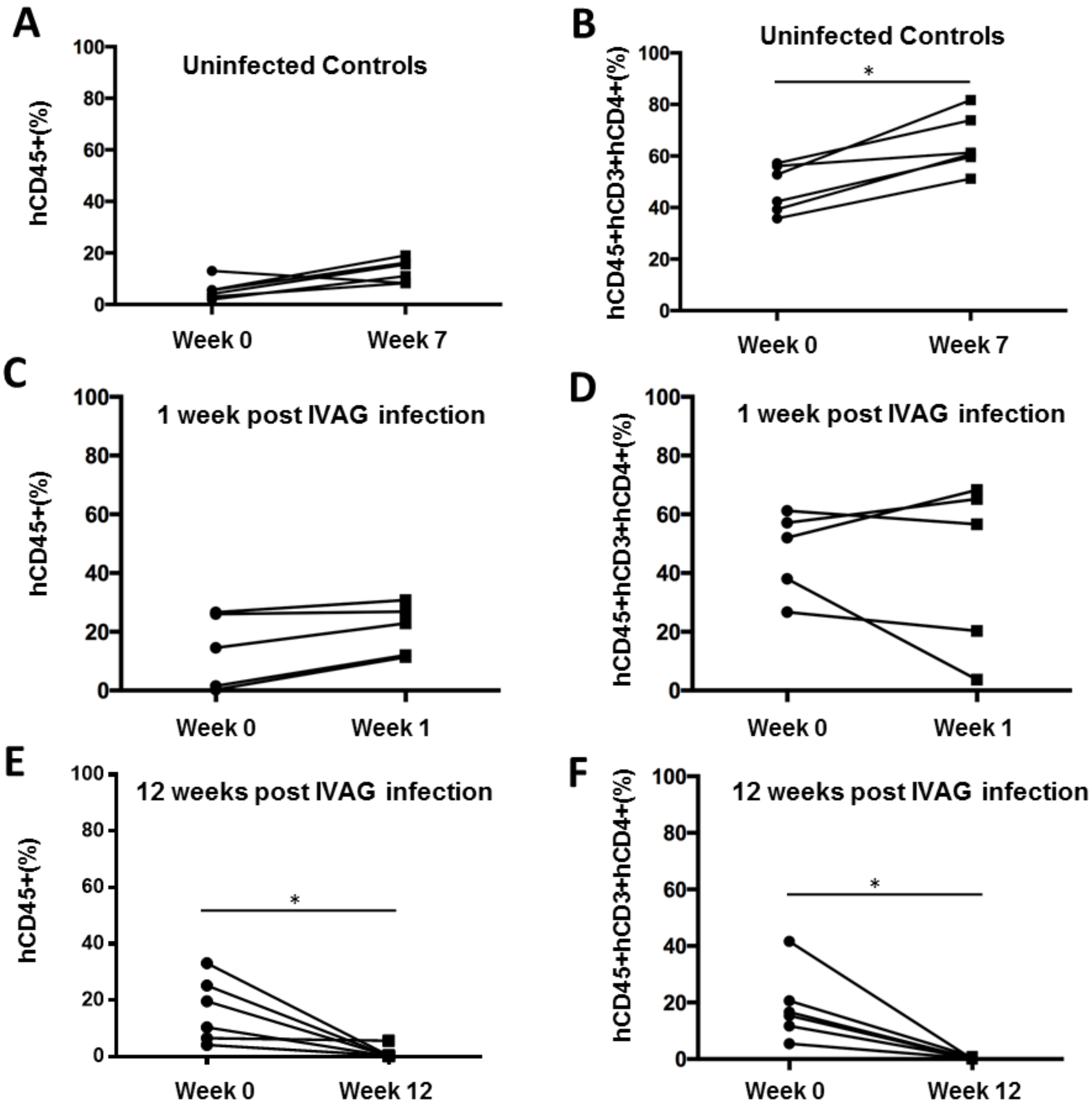

Supplementary Figure S5. Intravaginal HIV-1 infection in humanized mice leads to a decrease in circulating human leukocytes over time. Populations of leukocytes were quantified in the peripheral blood of uninfected (A, B), and infected (C-F) Hu-mice by flow cytometry. Hu-mice never exposed to HIV (controls, N=6) had stable proportions of circulating human CD45+ cells over 7 weeks (A), and a significant increase in the proportion of hCD45+hCD3+hCD4+ cells over the same time frame (B) (N=6; 5 Hu-mice <10% hCD45 and 1 Hu-mouse >10% hCD45, t-test). In Hu-mice infected IVAG with 10^5 TCID50 HIV-1 (NL4.3-BAL), the percentage of hCD45+ (C) and hCD45+hCD3+hCD4+ (D) cells remained stable at 1 week post-infection (N=5; 2 Hu-mice <10% hCD45 and 3 Hu-mice >10% hCD45, t-test). By 12 weeks post-infection, a significant decrease in hCD45+ (E) and hCD45+hCD3+hCD4+ cells (F) was observed (N=6; 2 Hu-mice <10% hCD45 and 4 Hu-mice >10% hCD45t-test). Week 0 was approximately 12-14 weeks following reconstitution with human immune cells. (*P<0.05).

	hCD45% in Hu-mice by Donor				
	Donor 1	Donor 2	Donor 3	Donor 4	Donor 5
	5.54	37.8	5.64	0.43	5.83
	1.21	6.45	0.71	0.88	7.68
	1.01	32.4	16.8	0.99	20.3
				1.59	17.2
**Average Reconstitution by Donor**	**2.59**	**25.55**	**7.72**	**0.77**	**11.27**

Supplementary Figure S6. Immune reconstitution does not depend on cord blood donor. The quantity of hCD45+ cells in the peripheral blood of Hu-mice does not appear to be dependent on the placental cord blood donor. Hu-mice with < and >10% hCD45 in the peripheral circulation can be derived from the same cord blood donor.
